# CCR5 and HIV Infection, a View from Brussels

**DOI:** 10.3389/fimmu.2015.00295

**Published:** 2015-06-08

**Authors:** Marc Parmentier

**Affiliations:** ^1^Welbio and IRIBHM, Université Libre de Bruxelles, Brussels, Belgium

**Keywords:** G protein-coupled receptors, chemokines, human immunodeficiency virus, CCR5, gene variants

HIV co-receptors were quite far from our main interests at the end of 1995, and we got involved in this field in a totally unexpected way. Our interest in chemokines was even relatively new at that moment, as we were mostly dealing with the characterization of new G protein-coupled receptors (GPCRs) in various areas such as endocrinology, neuroscience, and olfaction. Candidate receptors for leukocyte chemoattractant factors were part of an expanding repertoire of “orphan” receptors under study. I will essentially describe here a relatively short period of years 1995–1996, which has been one of the most hectic in my scientific career. This period is viewed from our perspective in Brussels, as I do not know for sure what was going on in other laboratories around the world, besides the results of these activities in terms of publications, communications in meetings, or personal contacts. I apologize in advance for the unavoidable bias in this sort of “historical” review.

A few years earlier, in the end of 1980s, our Institute had a strong focus on thyroid research. The most dynamic part of this activity was the cloning of some of the main actors of thyroid hormone biosynthesis, the hormone precursor thyroglobulin and the iodinating enzyme thyroperoxidase. A primary objective at that time was the cloning of the main regulator of thyroid function, the thyrotropin receptor, which was known as coupled to the stimulation of adenylate cyclase through the G_s_ protein. It is the search for the thyrotropin receptor that ultimately led, amongst many other unexpected findings, to our contribution to the characterization of CCR5 and its role in HIV infection.

G protein-coupled receptors constitute the largest family of membrane receptors and collectively play a major role in all physiological and pathophysiological processes. GPCRs share a common structural organization with seven transmembrane segments, and a common way of modulating cell functions by regulating effector systems through heterotrimeric G proteins and arrestins. The first GPCR sequences (rhodopsin, β-adrenergic, and M1 muscarinic receptors) were obtained in 1986–1988, following protein purification and peptide sequencing approaches. As a result, the common transmembrane organization and structural relatedness of GPCRs became obvious. Gilbert Vassart, leading the molecular biology group of the Institute, suggested applying the newly developed PCR method to the search of new members of the GPCR family, by using degenerate primers corresponding to the most conserved motifs among the small number of available GPCR sequences. A Ph.D. student in the Institute, Frédérick Libert, set up the procedure very successfully, and cloned within a few weeks, four new members of the GPCR family, that were referred to as “orphan” receptors (1). These were later characterized as CXCR7, serotonin 5HT1Dα, and adenosine A1 and A2a receptors. In the aftermath, a bunch of other orphan receptors were cloned, and we characterized the target of this new cloning strategy, the thyrotropin receptor (2). This PCR cloning approach, used first in Brussels, was applied broadly by other labs afterwards, and contributed significantly to the vigorous reporting of new GPCRs in the early 1990s.

In our hands, the first CCR5 sequences originated from a screen performed by Catherine Mollereau in early 1993 with the aim of identifying subtypes of opioid receptors. This screen led among others to the cloning of ORL1, a fourth member of the opiate receptor family, and the identification of its peptidic ligand nociceptin (3). A number of partial sequences were also similar to the first chemokine receptors, CXCR1, CXCR2, and CCR1, reported by the groups of Phil Murphy and Tom Schall (4, 5). We thus decided to engage into the functional characterization of these candidate chemokine receptors. The cDNA encoding CCR5 was expressed in CHO-K1 cells and tested in a microphysiometer, an ancestor of the “label free” instruments, which measured changes in cell metabolism by monitoring the acidification rate of the culture medium. MIP-1α/CCL3, MIP-1β/CCL4, and RANTES/CCL5 were identified by a French post-doc, Michel Samson, as three chemokines able to activate the receptor. The manuscript was first submitted to JBC in early September 1995, but was rejected after a 3-month reviewing process. It was resubmitted to Biochemistry in December (6).

In the meantime, a paper was published in December 1995 by the group of Paolo Lusso and Robert Gallo (7), describing that three chemokines, MIP-1α, MIP-1β, and RANTES, were able to inhibit infection of cells by macrophage-tropic HIV-1 strains. The link between the pharmacology of CCR5 and the profile of HIV inhibitory factors was of course striking. With no tools at hand for studying HIV, we first mailed Robert Gallo in January 1996 to propose some kind of collaboration to study the role of CCR5 in HIV infection. We never got an answer to this letter. It was quite clear at that time that we were not the only group to have CCR5 on hands. There were a bunch of very active groups in the chemokine receptor field, such as those of Philip Murphy, Craig Gerard, and Tom Schall. CCR3 and CCR4 had been published in late 1995 and Phil Murphy had reported the CCR3 sequence with MIP-1α, MIP-1β, and RANTES as agonists. This was later retracted as a result of a clone handling mistake, but it was quite clear that CCR5 and its pharmacology were in other hands as well.

While considering other potential collaborators, our manuscript dealing with CCR5 pharmacology became available, and very rapidly afterwards, I got a mail from Bob Doms in Philadelphia, proposing to join efforts on this topic. We sent to Bob plasmids encoding CCR5 and a set of related receptors we had at that time. Bob was obviously not alone in this game. In the HIV community, the existence of an HIV co-receptor, the orphan GPCR LESTR (and future CXCR4), for T-tropic HIV strains was already well known. The data would appear 1 month later in an April issue of Science (8). Many HIV groups were therefore looking for other GPCRs that would mediate the entry of HIV in macrophages and got in touch with teams involved in the chemokine receptor field. The race was fierce, and five papers reporting CCR5 as HIV co-receptor were published within a week in Nature, Cell, and Science in June 1996 (9–13). As a measure of the rush that took place in editorial offices and printing houses, our common paper with Bob Doms submitted on June 10 was published by Cell on June 28 with several pages printed upside down.

CCR5 seemed to play a key role in the entry of HIV strains involved in disease transmission. Soon after the first feed-back by Bob Doms of the experiments performed in Philadelphia, Gilbert Vassart suggested to check whether variants of CCR5 could be responsible for the variable susceptibility to HIV infection. We first obtained from a clinician of the nearby hospital, Claire Farber, DNA samples from three patients with slow disease progression and a few uninfected controls. Unexpectedly, Frédérick Libert and Michel Samson identified in this small series one slow progressor but also two control individuals as heterozygous for the same mutation of CCR5, a 32-base pair deletion in a region corresponding to the second extracellular loop of the receptor, and resulting in a frame shift and early termination (Figure 1). This mutant form of the CCR5 gene did not explain the slow progression of the patients tested. It was clear however that the resulting CCR5 mutant could not act as a functional receptor, and that the mutant allele was quite frequent. Within days, we sent a plasmid encoding this CCR5 mutant to Bob Doms for testing its function as HIV co-receptor, initiated experiments to demonstrate its deficiency as a chemokine receptor, and started collecting samples to study the frequency of the mutation at a larger scale. There were well-established cohorts of uninfected but multiply exposed individuals, but a few phone calls suggested to us that obtaining the genomic DNA from these cohorts would take ages compared to the pace at which this field was developing. We opted therefore for a more accessible approach. Starting from our local contacts in the campus hospital, were gathered within a week from various hospitals in Belgium and France, collections of DNA samples from cohorts of HIV-infected patients and uninfected controls, reasoning that the frequency of the mutant CCR5 allele should be different between these two groups if this allele was protective against HIV infection. We also collected DNA samples from about a hundred volunteers in the Institute’s staff. Testing these samples as they arrived builded progressively what is now known as the allele frequency of the Δ32 allele, around 10% in Western Europe. More importantly, while the number of homozygotes was in the expected range for Mendelian distribution in the uninfected group, there was a lack of homozygotes in the HIV-infected group. When each group reached over 700 individuals, the *p* value was below 0.0005. In the meantime, we had also found three Δ32 homozygotes within the institute personnel. We could rush blood cells to our Philadelphia collaborators to check whether these cells were indeed resistant to macrophage-tropic, but not T-tropic HIV-1 strains. This was indeed the case.

**Figure 1:**
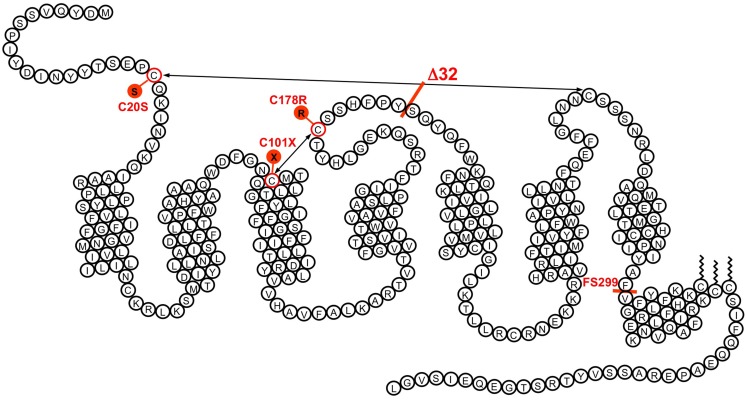
**The transmembrane organization of CCR5 is represented with its seven transmembrane segments, the eighth α-helix parallel to the plasma membrane, the two disulfide bonds, and the palmitoylated cysteines**. The position of some of the variants demonstrated to affect CCR5 function is indicated. The Δ32 mutant, with an average allele frequency of about 10% in European populations is the most frequent. Two missense mutations (C20S and C178R) affect one of the disulfide bonds necessary for the correct folding of the receptor. Two other mutations (C101X and the frame shift mutant FS299) result in early termination of translation. None of these mutant receptors is properly expressed at the surface of cells. Besides Δ32, the most frequent mutation is FS299, with an allele frequency of 2.9% in Chinese subpopulations. The allele frequencies of other mutations are well below 1%.

The manuscript was submitted to Nature in mid-July 1996. Although there was a strong interest of the Editor, one of the referees opposed us the fact that our cohorts were not constructed according to the rules. While we quite agreed on this, we had to fight to convince the editor that the data were clear enough to overcome weaknesses in cohort structure, and that there was no time to be spent on theoretical considerations. The final argument came when we could state that a concurrent manuscript had been submitted to Cell by the Ned Landau group and that it was being reviewed positively. As a result, we were requested to respond to the latest referee comments by correcting the text at the proof stage, and the two papers appeared in August 1996 (14, 15).

It was shown later on by various groups that protection by the Δ32 allele was not complete, and a few infected Δ32 homozygotes have been reported within the following years. In the following months and years, we have studied the structure–function relationships of CCR5 in relation to its role of chemokine receptor and HIV co-receptor, analyzed the distribution of the Δ32 mutation in various populations of the world, and tested the functional consequences of other, less frequent, variants and mutants of CCR5 (Figure 1). But somehow, the excitement was over, and subsequent research became more “routine.” The characterization of the CCR5 Δ32 mutation and its consequences on infection rate by HIV had validated CCR5 as an obvious target for the development of drugs targeting CCR5 and the entry of macrophage-tropic HIV strains. Many pharmaceutical companies, including Takeda, Pfizer, GSK, and Schering Plough, started immediately screening programs that resulted a few years later into CCR5 antagonists. While Takeda’s TAK779, GSK’s aplaviroc, and Schering Plough’s vicriviroc failed in clinical trials for toxicity reasons, Pfizer’s maraviroc went successfully through clinical testing and was approved in 2007 as the first-in-class CCR5 antagonist and HIV entry inhibitor. Overall, this has been a very short path (11 years altogether) between the discovery of a target and the launch of a small molecule in the clinics. With the present availability of fast and efficient mutagenesis techniques such as the CRISPR/Cas9 system, gene therapy approaches for inactivating CCR5 in the hematopoietic system are also being considered actively for the treatment of HIV infection.

## Conflict of Interest Statement

The author declares that the research was conducted in the absence of any commercial or financial relationships that could be construed as a potential conflict of interest.
